# Health Properties and Composition of Honeysuckle Berry *Lonicera caerulea* L. An Update on Recent Studies

**DOI:** 10.3390/molecules25030749

**Published:** 2020-02-09

**Authors:** Marta Gołba, Anna Sokół-Łętowska, Alicja Z. Kucharska

**Affiliations:** Department of Fruit, Vegetable and Plant Nutraceutical Technology, Wrocław University of Environmental and Life Sciences, Chełmońskiego 37, 51-630 Wrocław, Poland; anna.sokol-letowska@upwr.edu.pl (A.S.Ł.); alicja.kucharska@upwr.edu.pl (A.Z.K.)

**Keywords:** *Lonicera caerulea*, polyphenols, iridoids, anthocyanins, biological activity

## Abstract

*Lonicera caerulea* L., also known as haskap or honeysuckle berry, is a fruit commonly planted in eastern Europe, Canada and Asia. The fruit was registered as a traditional food from a third country under European Union regulations only on December 2018. It is resistant to cold, pests, various soil acidities and diseases. However, its attractiveness is associated mostly with its health properties. The fruit shows anticancer, anti-inflammatory, and antioxidant activity—important factors in improving health. These features result from the diverse content of phytochemicals in honeysuckle berries with high concentrations of phytocompounds, mainly hydroxycinnamic acids, hydroxybenzoic acids, flavanols, flavones, isoflavones, flavonols, flavanones and anthocyanins but also iridoids, present in the fruit in exceptional amounts. The content and health properties of the fruit were identified to be dependent on cultivar, genotype and the place of harvesting. Great potential benefits of this nutritious food are its ability to minimize the negative effects of UV radiation, diabetes mellitus and neurodegenerative diseases, and to exert hepato- and cardioprotective activity.

## 1. Introduction

**Figure 1:**
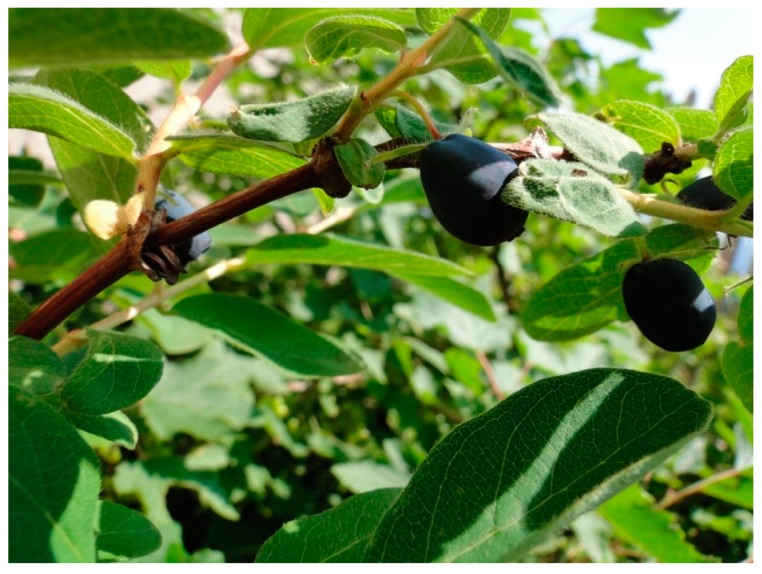
*Lonicera caerulea* L. with fruit.

*Lonicera caerulea* L. is a plant well-known in eastern countries, particularly in China and Russia [1]. It is commonly known as haskap berry (referred to Japanese blue honeysuckle type) or honeyberry (Russian and Kuril varieties of blue honeysuckle) [2], and to avoid redundancy, the name “honeysuckle” will be used further in the text to describe all varieties. The plant naturally occurs in forests in Europe, North Asia and North America, mostly in mountainous and low-lying wet regions [3]. Japanese Ainu aborigines have considered honeysuckle berries as an “elixir of life”, and on Hokkaido Island, a juice made out of the fruit is sold as a “gold remedy for eternal youth and longevity” [2,4]. In recent years, it has been widely planted in Europe—mainly in Poland, Slovenia, the Czech Republic and Slovakia [5]—but it is still gaining in popularity. Figure 1 presents a photograph of the plant. It belongs to the Caprifoliaceae family, and its fruit is known as honeysuckle berries (mostly referring to Japanese blue honeysuckle), also named honeysuckle berries (associated with the commercial name for Russian and Kuril cultivars) [2,6]. The genus *Lonicera* consists of over 200 cultivars [7]. Currently, the most commonly planted are honeysuckle berries originating in Russia, Japan, Canada and Poland. The cultivars most popular and easiest to grow are tundra, borealis, indigo gem, blue lightning and kamchatka [2]. A great advantage of the fruit is its high resistance to low temperatures. It can survive up to −40 °C and the flowers to −7 °C. Moreover, they are not affected by variations in soil pH, presence of pests or diseases [5]. The berries have an elongated elliptic or cylindrical shape, are dark purple in color and a waxy coating can be observed on their surface. Their weight ranges from 0.3 to 2.0 g. They can reach approximately 2 cm in length and 1 cm in width [6]. The taste can be characterized as bitter to sour-sweet, varying among cultivars [8]. 

The number of studies investigating the properties and content of berries is growing. The aim of this paper is to present the current state of art about the composition, health properties and recent findings regarding the plant.

## 2. Acid Composition in *Lonicera caerulea* L.

The berries show high content of numerous primary and secondary metabolites [5]. One of them is ascorbic acid, which plays an important role as an antioxidant agent, counteracting harmful processes such as inflammation. Honeysuckle berry is claimed to be one of the richest sources of vitamin C among all the berries. The content can vary based on climate and cultivation conditions, genotype, stage of ripeness and harvesting time [9,10,11]. The main environmental factors influencing ascorbic acid level are solar radiation, average temperature and precipitation [12]. It was shown that lower temperatures and wet weather have a positive impact on ascorbic acid content [9]. A study conducted by Jurikova et al. [13] proved that ascorbic acid values of certain honeysuckle berry cultivars can even exceed other fruit generally considered as rich in vitamin C. Oranges contain 31 mg/100 g of ascorbic acid, red currants from 35 to 90 mg/100 g, black elderberries around 30 mg/100 g and raspberries between 16 to 32 mg/100 g [5], while *Lonicera caerulea* cultivars show values up to 186 mg/100 g [13]. Another study indicates values ranging from 17 mg/100 g to 25 mg/100 g [3]. In a study performed by Viskelis et al. [14], it was found that berries planted in northern regions have higher levels of the acid than those from southern parts. It has been reported that consumption of vitamin C reduces reactive oxygen species levels, as well as triglyceride content, suggesting anti-inflammatory and anti-atherosclerotic properties [15].

Referring to organic acid composition, it is important to emphasize their importance for sensory quality of the fruit [5]. The amount of organic acids is negatively correlated with light intensity, contrary to the amount of accumulated sugars [16]. It was also reported that organic acid content was negatively correlated with total phenolics, meaning that acids decrease when the phenolic content rises [17]. The most prevalent organic acids found in honeysuckle berries are citric acid, which comprise 62% of total organic acids, malic acid (30%), quinic acid (6%), tartaric acid (1%), shikimic and fumaric acid [17]. These values correspond to the total organic acid content in honeysuckle berries of 6.55 to 8.85 mg/g, and the results are dependent on the fruit cultivation location. Another study presented values ranging from 6.75 to 11.04 mg/g [5]. The values in other berries exceed all these numbers; for bilberries, the reported amount of organic acid is equal to 1031 mg/100g, blackcurrants contain 2137 mg/100 g and red currants 1805 mg/100 g. A study performed in Slovenia initially compared honeysuckle berry composition of the cultivar aurora from different locations and also presented the results of various cultivars from one region. There were found significant differences between the regions, where the highest values reached 8.86 mg/g and the lowest 6.56 mg/g. The discrepancies between cultivars ranged from 11.04 mg/g for borealis to 6.75 mg/g for aurora.

## 3. Sugar and Mineral Content

Fructose comprises more than half (55%) of total sugars found in honeysuckle berries [5]. Other sugars present in the fruit are glucose (43%) and sucrose (3%). The fruit is considered to contain less sugar than, for example, blueberries and could be recommended for a diabetic diet. The total sugar content in honeysuckle berries ranges from approximately 15 mg/g up to 25.85 mg/g of fresh fruit, while the value for blueberries is 78.1 mg/g, for raspberry 45.5 mg/g and for red currant 38.2 mg/g [5,16,17]. Higher light exposure and later, harvesting time, positively correlate with sugar content [18,19]. One cultivar that is low in sugar is honeybee, containing 15.57 mg/g, while higher levels can be observed in tundra (17.34 mg/g) and aurora (25.85 mg/g) [5]. The impact of irrigation on sugar levels was analyzed, but no significant correlation was observed [20].

Considering mineral content, honeysuckle berries contain similar amounts of potassium, calcium and magnesium to wild berries [21]. Potassium is the most prevalent compound found in the berries, followed by phosphorus, magnesium and calcium, while sodium shows the lowest concentration [20]. The content of the mentioned minerals can vary a lot. A study by Plekhanova et al. [22] indicated values ranging between 300 and 500 mg/kg fresh weight (FW). Another study described potassium levels ranging between 10 and 15 mg/kg [20], while Sochor et al. [23] reported potassium levels in honeysuckle berries ranging from 3000 to 5000 mg/kg. Species, as well as the year of plant vegetation, was found to be a significant factor corresponding to potassium level in the fruit [20]. Magnesium content shows values between 79 and 163 mg/kg [23], while the average result in the study by Jurikova and Matuskovic [20] indicated the content of 711 mg/kg. The mean value of phosphorus in the latter study was 2252 mg/kg, while another source reported an average value of 486 mg/kg [23]. The level of calcium was, on average, 1077 mg/kg and, for sodium, 81.67 mg/kg [20], and according to another study, it showed values of 442 mg/kg and 14 mg/kg for calcium and sodium, respectively [23]. Lower mineral values may result from discrepancies in cultivation conditions and the impact of climate variation. Minerals play an important role in processes such as nerve impulses transmittance, hormones formation and heartbeat regulation. Moreover, they are also a structural part of numerous enzymes and are also among the key factors responsible for functioning of immune and brain systems [24].

## 4. Phytocompound Composition in *Lonicera caerulea* L. Fruit

It was reported that honeysuckle berries show the highest level of polyphenolic compounds out of all the berries grown in Canada [25,26]. The amount identified in one of the studies (1111.17 mg/100 g), exceeds even the values identified in bilberries (778.47 mg/100 g), wild strawberries (437.04 mg/100 g) and purple raspberries (638.09 mg/100 g) [26]. Phenolics exert a great impact on health, and thus, attract a lot of attention and have been a subject of many studies. The total phenolic content (TPC) reaches values from 1.41 to 11.42 mg gallic acid equivalents (GAE) per gram of FW. Studies have shown that honeysuckle berry fruit contains triterpenoic acids, β-carotene, catechol, flavonols, chlorogenic acid and many more acids [27]. The phenolic compounds can be classified into nine groups: hydroxycinnamic acids, hydroxybenzoic acids, flavanols, flavones, isoflavones, flavonols, flavanones and anthocyanins [5]. Out of all phenolics found in honeysuckle berry fruit, the greatest proportion, ranging between 36% and 51%, is observed for anthocyanins—peonidin glycosides, cyanidin and petunidin. The main anthocyanin identified was cyanidin 3-*O*-glucoside [28,29]. The level of anthocyanins is claimed to be dependent on harvest time and cultivar [11]. Moreover, it was reported that another factor influencing the anthocyanin level is low temperature [5]. Higher content of anthocyanins could result from low temperatures and solar radiation. It was also observed that overripe berries show higher concentrations of anthocyanins [30,31]. Larger fruits were found to contain more anthocyanins due to the high area of the skin surface, where the compounds mostly accumulate [5]. The second most abundant compounds were phenolic acids (24%). Phenolic acids can be divided into two classes according to their structure: derivatives of cinnamic acid and benzoic acid [32]. A study by Zadernowski et al. [33] showed that 61.1% of honeysuckle berry total phenolic acids consisted of hydroxycinnamic acids (mostly chlorogenic acid) and their derivatives (*p*-coumaric acid and *m*-coumaric acid). Another study reported the abundance of rosmarinic, vanillic and gentisic acids, with values of 0.08% in total [34]. Flavonols—kaempferol, isorhamnetin glycosides and quercetin—correspond to 22% of total phenolics. Quercetin 3-*O*-rutinoside showed the highest contribution to total flavonols. Similar to anthocyanin content, flavonol levels are also positively correlated with higher altitudes. A study by Albert et al. [35] indicated an increase of flavonol concentration with declining temperature. Similar to the content of anthocyanins, the level of chlorogenic acid and derivatives depends on cultivation localization and the cultivar [36]. It was also reported that hydroxycinnamic acid levels positively correlate with higher altitude, being associated with a response to stronger UV radiation observed there [5,17,37]. A strong negative correlation was observed between the weight of the fruit and total hydroxycinnamic acids, hydroxybenzoic acids, flavanones and flavanols [5]. The total phenolic content is negatively correlated with organic acids but shows a positive correlation with sugars. Flavanols were one of the least prevalent substances found in honeysuckle berry (11% of total phenolics). Their content is also UV radiation-dependent. Flavanols consisting of (−)-epicatechin, (+)-catechin and procyanidin oligomers can be found mostly in small, nonmature fruit, and their content shows no correlation with temperature or altitude. However, a strong correlation with fruit weight can be observed. Another potential influencing factor is exposure to light. Flavones which are also present in honeysuckle berry fruit include luteolin glycosides, which show increased levels with the time of harvesting [11]. According to the literature, a long-term consumption of polyphenol-rich products protects against cardiovascular diseases, osteoporosis, lung damage, certain types of cancers, type 2 diabetes, gastrointestinal disorders and neurodegenerative diseases [38]. 

A comparison of total phenolics was carried out by Ochmian et al. [11], who investigated the impact of harvesting time on the fruit. The results showed that, in both analyzed cultivars, the total phenolic content was higher at the end of the harvesting time. In this study, anthocyanins were clearly dominating compounds, with the major constituent being cyanidin 3-*O*-glucoside. In the research of Senica et al. [5], four cultivars and their phenolic contents were compared. The highest total phenolic content was reported in tundra, with 268.22 mg/100 g, followed by honey bee, with 225.30 mg/100 g. A slightly lower value was recorded in the cultivar aurora, with 219.64 mg/100 g, and the lowest content was observed in borealis, with 173.51 mg/100 g. The same research also emphasized the presence of saponins. This compound is mostly present in exotic fruits such as papaya, honey locus and dragon fruit, but it was also found in honeysuckle berries. Its level was reported to be positively correlated with ascorbic acid. The highest saponin content out of all cultivars analyzed in the study was shown by honey bee, with 640.79 mg of diosgenin equivalents/100 g. 

Tannins are another group of compounds found in honeysuckle berries. They are related to astringent taste and are present in high concentrations in pomegranate peel (29,223 mg GAE/100 g) and persimmon (1010 mg GAE/ 100 g). The study of Senica [5] identified tannin content in the berries to range from 129.81 mg GAE/100 g to only trace amounts, depending on the cultivar. It is also worth emphasizing the presence of iridoids, which are not commonly found in fruits. They belong to a group of monoterpenoids; in terms of structure, they are cyclopentan-[C]-pyran monoterpenoids, and they provide a link between alkaloids and terpenes [39,40]. They are responsible for the bitter taste in fruit and possess a wide range of antioxidant and anti-inflammatory properties [37,41]. In a study by Kucharska et al. [42], there were fifteen iridoids identified. The research reported that even though the 27 cultivars which were analyzed show similar content of most phenolic compounds, the profile of iridoids varies significantly, ranging from the minimal amount of 119.95 mg/100 g of FW in the cultivar dlinnoplodnaya, to maximally 276.43 mg/100 g of FW identified in the berry smart blue cultivar. In general, the high level of phytocompounds and diverse composition correspond to high nutraceutical values and a high health potential of honeysuckle berries.

## 5. Antioxidant Activity

High antioxidant activity has become a topic of numerous studies. Consumption of products containing high levels of antioxidants shows a positive effect on counteracting cancer tumor and inflammatory diseases [43]. It was identified that honeysuckle berry serves as a rich source of free radical scavengers. According to the study by Halvorsen et al. [44], blackberries, strawberries, raspberries, cranberries and blueberries are among the top antioxidant foods. Rupasinghe et al. [25] in an in vitro test proved that honeysuckle berries show higher activity than commonly eaten strawberries or blackberries. According to the result of FRAP (ferric reducing antioxidant power) analysis, the antioxidant capacity of cultivar borealis is equal to 46.38 mg GAE/100 g FW, while the value for strawberries is 8.00 mg GAE/100 g FW and for blackberries 15.03 mg GAE/100 g FW. The activity can vary based mainly on the cultivar and genotype, which could influence the content quality and quantity [42]. Among the most antioxidative compounds of berries are vitamin C and polyphenols, mainly anthocyanins, phenolic acids, flavonols and flavanols [45,46]. Rop et al. [47] found that the antioxidant activity can vary significantly among species. The highest values out of twelve analyzed cultivars were identified in Zolushka and Gerda (10.17 and 9.92 g of ascorbic acid equivalent (AAE)/kg fresh mass, respectively), while the lowest levels were observed in leningradskii velikan and nimfa (6.59 and 6.75 g of AAE/kg FW, respectively). The results are consistent with those obtained by Sochor et al. [23], who also examined similar cultivars. Their study showed the zolushka cultivar to be the best antioxidant and to contain the most phenolic compounds. Compared to other commonly consumed fruits, these values are considerably high. The results of antioxidant activity of cherries reach a maximum value of 0.9 g of AAE/kg FW, while, for plums, it is up to 6 g of AAE/kg FW [47,48]. Rupasinghe et al. [25] took into account other cultivars, and they observed the highest antioxidant capacity in borealis, indigo gem and tundra. Borealis showed the strongest activity, as well as having the highest total phenolic and total flavonoid contents determined by spectrophotometric methods. Cultivars amur and jolanta were reported to be among other strong antioxidative agents [42]. Less active ones include atut and karina. There was also reported a strong correlation between antioxidant power and anthocyanins. In contrast, the amount of iridoids is weakly correlated with antioxidant capacity. This discrepancy can be explained by the presence of OH groups, which are able to reduce free radicals [49]. Therefore, iridoids could serve more as an anti-inflammatory or antibacterial agent, which gives honeysuckle berries even wider health properties. Moreover, a study by Zhao et al. [50] revealed that the antioxidant activity depends on the way of bioactive compounds’ extraction. According to the study, methanol extracts show higher antioxidant activities than those obtained with ethyl acetate or dichloromethane. An in vivo study reported that phenolic extracts from honeysuckle berry increased levels of reduced glutathione strictly connected with antioxidant activity [51]. A summary of total content of polyphenols, anthocyanins, flavanols, phenolic acids and iridoids in 19 cultivars, obtained from various studies, is presented in Table 1. All the conducted research has confirmed the high antioxidant activity of honeysuckle berries, depending mostly on the cultivar and, thus, the content of bioactive compounds.

## 6. Other Health Properties

The plant is commonly used in traditional medicine, especially in Japan, being exceptional due to its anti-ageing properties, as well as protecting against heart diseases and gastrointestinal problems [1]. Scientific studies have confirmed cardio- and neuroprotective, anticancer and anti-inflammatory activity of the honeysuckle fruit [52]. Moreover, antimicrobial and antidiabetic properties have been observed. The mentioned features are mostly connected to bioactive compounds present in the berries, mainly phenolic compounds including anthocyanins, chlorogenic acid and quercetin [42]. The presence of iridoids in honeysuckle berries also corresponds to its attractiveness, as they are not commonly found in fruit. Apart from honeysuckle, the compounds can only be found in cornelian cherry fruits, bilberry and cranberry. Prevalent antioxidants are able to reduce reactive oxygen species, counteracting aging processes [53]. Moreover, according to conducted studies, regular consumption of berries could reduce cancer and insulin resistance, minimize bone loss and improve neurocognitive functions [54,55,56,57]. Studies have also shown the positive impact of honeysuckle berries on inhibiting melanogenesis, resulting in a whitening effect [1]. 

### 6.1. Antimicrobial Properties

Bacterial tests have identified antimicrobial properties of honeysuckle berries, being particularly efficient against *Kocuria rhizophila*, *Bacillus subtilis* and *Campylobacter jejuni* but without affecting health-positive bacteria, meaning the berries could serve as a probiotic food [1,58]. It was also proved that extracts of freeze-dried berries and phenolic extracts can effectively counteract microbial adhesion, and thus, prevent various infections such as mouth and urinary diseases [59]. The extracts reduced adhesion of *Staphylococcus epidermidis* (zero colony forming units (CFU)); *Escherichia coli* (4.55 × 10^3^ CFU when freeze-dried extract was used and 1.45 × 10^1^ CFU when phenolic extract was used); *Streptococcus mutans* (4.48 × 10^1^ CFU and 0 CFU, respectively) and *Enterococcus faecalis* (6.9 × 10^3^ CFU and 4.5 × 10^0^ CFU, respectively), in comparison to the control samples (2.59 × 10^3^, 1.6 × 10^4^, 5.44 × 10^4^ and 6.1 × 10^4^ CFU) [59].

### 6.2. Neuroprotective Activity

It has been reported that daily intake of polyphenols (prevalent in honeysuckle berries) minimizes risk of dementia, neurodegenerative diseases and stroke mainly through free radical scavenging, activation of survival genes and signaling cascades, transition metal chelation, modification of neuroinflammation and regulation of mitochondrial function [60]. Numerous studies have focused on the impact of blue berries, citrus fruit and green tea on human health. It was proved that blueberry extract is able to significantly improve episodic memory and visuospatial working memory as a result of three-months supplementation and can also reduce systolic blood pressure level already after three-months supplementation [61]. A recent study by Bell and Williams [62] demonstrated a significant positive influence of honeysuckle berry extract on physiological and cognitive functions in the immediate postprandial period. A 400 mg dose could lower diastolic blood pressure and heart rate already after 1.5 h after administration. This observation was related to already examined properties of the vasodilatory and glucoregulatory impact of anthocyanins [63,64]. The mentioned physiological changes can be associated with increased cognitive function achieved through increased blood flow to the brain and higher glucose uptake to the brain. Administration of a 400 mg high dose of honeysuckle extract resulted in improvement of episodic memory. It was also suggested that lower doses of 100 mg are not sufficient to affect episodic memory. A limited impact of the extracts on working memory or executive function was reported. Based on all the study findings, honeysuckle berries have a great potential in terms of age-related memory deficits and are able to improve metabolic and vascular health.

### 6.3. Hepatoprotective, Cardioprotective and Radioprotective Effect

A cell culture test indicated that phenolic extracts from honeysuckle berries are able to inhibit liver microsome peroxidation and slow down low-density lipoprotein (LDL) oxidation [59]. This is a crucial factor in minimizing cardiovascular diseases such as atherosclerosis [65]. Tests conducted on mice revealed that phenolic extract of berries can minimize destructive effects of UV radiation—DNA breakage, keratinocytes and membrane damage [51]. Therefore, honeysuckle fruit can also serve as a potential radioprotective agent. A study also identified lower caspase-3 and caspase-9 activity, which are associated with reversing cellular apoptosis [66]. Moreover, research has shown that honeysuckle berry extracts protect DNA from damage, thus preventing carcinogenesis [53]. It has also been proved that cancer cells tend to enter apoptosis upon exposure to the berry extract [25].

### 6.4. Antidiabetic Properties

Diabetes mellitus (DM) is also a disease closely associated with a negative impact of free radicals. It is becoming more and more prevalent, with 451 million people affected worldwide in 2017, and it is estimated that 693 million individuals will be affected by 2045 [67]. It has been considered a health pandemic issue resulting in a high mortality rate and numerous complications [68]. Therefore, there is a high demand to find a minimally invasive treatment. A study by Sharma et al. [69] analyzed mice on a high-fat diet (HFD), which showed increased insulin, blood glucose, glycated hemoglobin (HbA1c), blood urea nitrogen (BUN) and creatinine levels. The mice also showed more degenerative lesions and a higher number of pancreas islet cells responsible for insulin/glucagon production. When mice were fed with honeysuckle berry extracts, the mentioned complications were significantly inhibited. Moreover, feeding with 400 mg/kg extracts resulted in inhibition of type II diabetes and other positive effects, contrary to HFD mice. The fruit was also considered to be a promising treatment for diabetic nephropathies. The study also confirmed dose-dependent activity of honeysuckle berries, which reveals a potential to be a medicinal food for diabetes treatment.

### 6.5. Anti-Inflammatory Properties

Free radicals’ activity can lead to inflammation, cancer and diabetes [70,71]. Moreover, chronic inflammation can cause metabolic diseases, which currently are a very common disorder [72]. Inflammatory pathways are mediated by different factors and enzymes, such as tumor necrosis factor (TNF-α), interferon-gamma (IFN-γ) and cyclooxygenase (COX-2). A healthy diet is one of a few agents influencing metabolic syndrome [73]. Therefore, there is a strong demand for natural plant-derived nutraceuticals aimed at counteracting the disease. Rich in polyphenols, honeysuckle berry shows a potential to scavenge free radicals and also demonstrated a potential to modify the TNF. A study by Rupasinghe et al. [74] presented a negative correlation between polyphenol content of honeysuckle extracts and proinflammatory cytokines COX-2, TNF-α, interleukin-6 (IL-6) and prostaglandin (PGE_2_). Inhibition of the inflammatory process was shown to be mostly effective at the start of the signaling cascade; thus, it can be assumed that honeysuckle berry extracts are more efficient in preventing the inflammation rather than treatment. When fibroblast cells were incubated with fruit extracts, a decrease in reactive oxygen species was observed, as well as release of inflammatory markers [75]. Gut microbiota is closely related to metabolic diseases, and gut dysbiosis is associated with metabolic syndromes, including diabetes [76,77]. A high-fat diet increases the level of endotoxins and increases serum levels of inflammatory cytokines connected with nonalcoholic fatty liver disease (NAFLD). Administration of honeysuckle berry polyphenols decreased both endotoxin and cytokines levels [78]. These results suggest that the berries could have an impact on gut bacteria, and thus, rebalance microecological conditions. NAFLD is also claimed to be associated with a reduced amount of *Bacteroidetes* [79,80]. Similarly, obese people have a higher *Firmicutes/Bacteroidetes* ratio than people with normal weight. Supplementation with honeysuckle berry-derived polyphenols increased the number of *Bacteroidetes* and reduced the amount of *Firmicutes*, resulting in a lower *Firmicutes/Bacteroidetes* ratio, which was observed to be dose-dependent [78]. 

Another very important aspect is the ability of honeysuckle berry to decrease eye inflammation, proved on rat models with uveitis [81]. Another group of rats fed with an unbalanced high fructose diet showed a decreased plasma lipid concentration and normalized triglyceride levels after supplementing honeysuckle extracts [82]. Based on the obtained results, the daily intake of phenolic extract beneficial for health promotion for an adult weighing 70 kg was calculated to be 0.8 g. Unfortunately, so far, not many in vivo studies have been conducted, so the exact impact on the human organism is not known.

The main reviewed properties and the concentrations of berries used are summarized in Table 2.

## 7. Food Products

Today, the products available on the market containing honeysuckle fruit are mostly jams, wines, candies, jellies, gelatin, ice cream and yoghurt [84]. A crucial advantage of honeysuckle juice is that its color is stable through time [7]. A promising solution to incorporate honeysuckle berries into a daily diet is using encapsulation techniques and creating value-added products [1]. The mentioned form would provide highly concentrated functional products of controlled substance release, high extract stability and the ability to target desired organs. There is still a strong need to develop such products. A breakthrough in terms of popularization of the honeysuckle berry occurred in 2018. The fruit was registered as a traditional food from a third country under European Union regulations on December 2018 [85]. A recent study by Senica et al. [86] compared different forms of honeysuckle berry products: infusion, juice, spread and smoothie. A spread made out of the fruit showed the highest content of ascorbic acid and phenolic compounds—302.02 mg/100 g of dry weight and 1753.54 mg/100 g, respectively. High values were also observed for the liqueur and smoothie, while in the infusion (fruit tea) and juice, the content of ascorbic acid was only 119.17 and 118.17 mg/100 g, respectively, and the phenolic content was 1138.75 and 1108.25 mg/100 g, respectively. All the mentioned products had a low sugar content and, therefore, can be served to diabetic patients.

## 8. Conclusions

*Lonicera caerulea* L. shows high health potential and is a promising source of numerous bioactive compounds, mainly anthocyanins, phenolic acids and flavonols. A unique feature of honeysuckle berries is the presence of iridoids, which are a great anti-inflammatory and antioxidant agent. The rich content of berries corresponds to numerous health benefits. Both in vitro and in vivo studies confirm efficient free radical scavenging of honeysuckle extracts. Reduction of reactive oxygen species is the main reason for properties such as antitumor activity, minimizing insulin resistance and neurocognition improvement. Recent studies emphasize the positive impact on the prevention of diabetes mellitus and decreasing the negative effects. Analysis of the exact content and mechanism of action of the extracts has attracted the attention of many researchers. Therefore, it has been confirmed that a daily intake of honeysuckle berries is able to improve physiological and cognitive functions. Developing honeysuckle-based products, a valuable source of health-promoting compounds which could serve as nutraceuticals, is an important aspect of current research. Definitely, the knowledge about the fruit is broadening, and it is worth continuously updating the topic.

## Figures and Tables

**Table 1:** Different polyphenol contents in analyzed cultivars of honeysuckle berry (ND—no data and FW—fresh weight).

Cultivar	Total Polyphenols (mg gallic acid/100 g FW)	Total Anthocyanins (mg/100 g FW)	Total Flavanols (mg/100 g FW)	Total Phenolic Acids (mg/100 g FW)	Total Iridoids (mg/100 g FW)	References
Amfora	563.2 ± 9.4 ^1^	471.3	26.3	48.0	232.0	[4,23]
Amur	732.6 ± 11.1	655.2	28.7	62.4	259.9	[4,23]
Aurora	219.6 ± 19.2	112.37 ± 6.7	8.15 ± 4.3	20.5 ± 9.7	0.5 ± 0.1	[5]
Borealis	173.5 ± 17.5	86.0 ± 7.0	19.4 ± 1.4	38.7 ± 4.3	0.3 ± 0.1	[5]
Fialka	659.6 ± 7.1	432.5	21.1	79.1	208.7	[4,23,47]
Gerda	823.6 ± 10.5	ND	ND	ND	ND	[23,47]
Goluboje vreteno	865.9 ± 10.9	273.8	36.1	65.2	197.5	[4,23,47]
Honey Bee	225.3 ± 19.3	86.3 ± 8.1	24.2 ± 3.5	51.7 ± 7.8	0.3 ± 0.1	[5]
Kamchadalka	779.8 ± 8.8	224.6	31.3	91.0	203.6	[4,23]
Leningradskij velikan	623.5 ± 8.8	220.3	24.6	61.0	217.6	[4,23,47]
Morena	623.5 ± 7.7	473.7	24.6	65.6	272.6	[4,23,47]
Nimfa	625.9 ± 5.4	557.7	18.3	62.2	158.5	[4,23,47]
Roksana	789.6 ± 10.3	197.3	39.2	71.1	150.0	[4,23,47]
Sinogalaska	778.9 ± 12.4	306.3	47.7	76.8	122.7	[4,23]
Vasilevskaya	698.5 ± 13.2	224.8	50.7	85.3	177.9	[4,23,47]
Viola	715.9 ± 8.4	151.7	32.6	59.0	150.3	[4,23,47]
Tomichka	825.9 ± 12.9	195.5	28.4	115.5	125.1	[4,23,47]
Tundra	268.2 ± 3.5	112.5 ± 2.4	24.0 ± 1.1	68.1 ± 2.4	0.4 ± 0.1	[5]
Zoloushka	856.9 ± 11.5	ND	ND	ND	ND	[23,47]

^1^ Data are presented as mean value ± standard deviation (SD).

**Table 2:** Health-associated effects of honeysuckle berries based on in vitro and in vivo tests.

Effect	Material Used in the Study	Method	Study Outcome	References
Improvement of physiological and cognitive functions.	Extract containing 400 mg anthocyanins	Double-blind, counterbalance, crossover intervention study.	Improvement of episodic memory and blood pressure after acute supplementation with honeysuckle berry extract.	[62]
Minimize negative effect of UV radiation—DNA breakage, keratinocytes and membrane damage.	10 and 25 mg/L extract	Solar stimulation for keratinocytes damage and impact of pre- and post-treatment with phenolic extract.	Suppression of UVB-caused injury of keratinocytes and decrease in reactive oxygen and nitrogen species (RONS).	[66]
Decreased eye inflammation.	100 μg/mL extract	Mice injected with lipopolysaccharide and honeysuckle berry extract.	Reduced level of nitric oxide (NO) and tumor necrosis factor (TNF- α).	[81]
Lowering plasma lipids and normalizing triglyceride levels for a positive effect on diabetes.	Extract containing 327 mg anthocyanins/g	Mice on cornstarch or high-fructose diet, with and without addition of honeysuckle berry extract.	Extract supplemented to an unbalanced diet ameliorated the disturbances in glucose and lipid metabolism.	[82]
Minimizing nonalcoholic fatty liver disease (NAFLD) and balancing gut microbiota dysbiosis.	250 g/L extract	Mice on high-fat diet fed with honeysuckle extract.	Attenuation of inflammation in NAFLD through modulation of gut microbiota.	[78]
Improve hepatic steatosis and insulin resistance.	250 g/L extract	Mice on high-fat diet containing honeysuckle berry extract.	Suppression of induced obesity and fat deposition, increased insulin sensitivity and attenuation of oxidative stress.	[80]
Decreasing insulin, blood glucose, glycated hemoglobin (HbA1c), blood urea nitrogen (BUN), creatinine level, inhibition of type II diabetes andpotential treatment of diabetic nephropathies.	400 mg/kg body mass extract	High-fat diet-induced mild diabetic mice administered with honeysuckle berry extract for 12 weeks.	Ameliorated diabetic and related complications in a dose-dependent manner.	[69]
Anti-inflammatory properties.	200 g/L extract	Human monocytes (THP-1) differentiated macrophages incubated with extracts.	Negative correlations between polyphenol concentration and proinflammatory cytokines.	[74]
Inhibition of liver microsome peroxidation and slowing down low-density lipoprotein (LDL) oxidation.	Phenolic extract of 18.5% anthocyanins	Rat hepatocytes incubated with extract.	Prevention of oxidation.	[83]
Antimicrobial properties.	ND	*Candida parapsilosis, Staphylococcus epidermidis, Escherichia coli, Enterococcus faecalis* and *Streptococcus mutans* exposed to extracts.	Superoxide scavenging activity and suppression of biofilm formation.	[59]
